# Zn^2+^ Uptake in *Streptococcus pyogenes*: Characterization of *adcA *and *lmb* Null Mutants

**DOI:** 10.1371/journal.pone.0152835

**Published:** 2016-03-31

**Authors:** Vittorio Tedde, Roberto Rosini, Cesira L. Galeotti

**Affiliations:** 1 Research Centre, GlaxoSmithKline Vaccines S.r.l., Siena, Italy; 2 Department of Medical Biotechnologies, University of Siena, Siena, Italy; Ross University School of Medicine, DOMINICA

## Abstract

An effective regulation of metal ion homeostasis is essential for the growth of microorganisms in any environment and in pathogenic bacteria is strongly associated with their ability to invade and colonise their hosts. To gain a better insight into zinc acquisition in Group A Streptococcus (GAS) we characterized null deletion mutants of the *adcA* and *lmb* genes of *Streptococcus pyogenes* strain MGAS5005 encoding the orthologues of AdcA and AdcAII, the two surface lipoproteins with partly redundant roles in zinc homeostasis in *Streptococcus pneumoniae*. Null *adcA* and *lmb* mutants were analysed for their capability to grow in zinc-depleted conditions and were found to be more susceptible to zinc starvation, a phenotype that could be rescued by the addition of Zn^2+^ ions to the growth medium. Expression of AdcA, Lmb and HtpA, the polyhistidine triad protein encoded by the gene adjacent to *lmb*, during growth under conditions of limited zinc availability was examined by Western blot analysis in wild type and null mutant strains. In the wild type strain, AdcA was always present with little variation in expression levels between conditions of excess or limited zinc availability. In contrast, Lmb and HtpA were expressed at detectable levels only during growth in the presence of low zinc concentrations or in the null *adcA* mutant, when expression of *lmb* is required to compensate for the lack of *adcA* expression. In the latter case, Lmb and HtpA were overexpressed by several fold, thus indicating that also in GAS AdcA is a zinc-specific importer and, although it shares this function with Lmb, the two substrate-binding proteins do not show fully overlapping roles in zinc homeostasis.

## Introduction

Zinc is the second most abundant transition metal in biological systems [1] [2]. In general, transition metals are key components of nearly 50% of all known enzymes with the dual role of being important structural elements as well as essential cofactors to an extensive range of enzymatic activities [3]. Specifically, zinc ions are present in many proteins involved in fundamental biological tasks, such as DNA polymerases, proteases, ribosomal proteins. However, despite its essential role in biology, Zn^2+^ can become toxic if accumulated to excess, as it competes with other metals for binding to active sites of enzymes thus disrupting normal metabolic processes. Hence, Zn^2+^ homeostasis in bacteria must rely on tightly regulated import and export mechanisms [4].

Two transport systems of importance in this context are the orthologous ATP binding cassette (ABC) transporters ZnuABC of *Escherichia coli* [5] and AdcABC of *Streptococcus pneumoniae* [6]. Acquisition of zinc from the environment is achieved by both transporters through their substrate-binding protein (SBP) component, a lipoprotein able to bind and transfer Zn^2+^ ions to the membrane-bound permease component of the transporter. In *Treponema pallidum* Zn^2+^-binding SBPs belong to two types, one showing promiscuous preference for metal binding (TroA) and the other specific for Zn^2+^ (ZnuA) [7]. Similarly, *S*. *pneumoniae*, as most streptococci, possesses two Zn^2+^-binding SBPs, AdcA and AdcAII, whose only partially redundant function in Zn^2+^ acquisition has been recently well characterized [8]. AdcA is more closely related to the high affinity zinc-specific ZnuA, typical of Gram-negative SBPs, although it contains significant structural differences. One such difference is the presence of an extended loop of ~200 amino acids at its C terminus containing a second Zn^2+^-binding domain with homology to the zinc-chaperone ZinT of Gram-negative bacteria [8]. AdcAII lacks this region and contains only the canonical amino-terminal Zn^2+^-binding domain [9]. Moreover, AdcAII requires the presence of polyhistidine triad proteins for the uptake of zinc *in vivo* [10], while AdcA can efficiently recruit zinc in the absence of Pht proteins [8].

Zinc homeostasis is crucial in any normal metabolic condition, however, it becomes particularly critical during infection, when both host and pathogen compete for the same essential metals. In addition, vertebrates have evolved defensive systems that deplete or flood infection sites with metal ions in order to eradicate the pathogen [11] [12]. Thus, during the most critical phases of their life cycle, namely interaction with the host, pathogens need the finest tuning of their metal homeostasis systems. A thorough characterization of all the components with a role in metal homeostasis in pathogenic bacteria would help considerably in providing an understanding of the mechanisms underlying bacterial infections. In this regard, recent work has characterized the role of two key components of the zinc efflux system in Group A *Streptococcus* (GAS), CzcD and GczA, demonstrating that zinc is crucial to the innate immunity response against GAS infection [13]. More recently, it has also been shown that zinc exerts its toxicity in GAS through inhibition of the central carbon metabolism and by causing disruption of capsule biosynthesis[14]. The essential role of zinc during infection had already be inferred in a previous report that describes how a GAS mutant that lacks Lsp, the only zinc receptor of GAS that has been structurally characterized[15], is attenuated in a murine subcutaneous ulcer model of infection[16].

Herein we report an attempt to gain a better insight into zinc acquisition in Group A *Streptococcus* by characterizing the orthologues of *S*. *pneumoniae* AdcA and AdcAII, named AdcA and Lmb, respectively, in *Streptococcus pyogenes* serotype M1 strain MGAS5005 [17]. It should be noted that Lmb has been previously named also Lsp, for lipoprotein of *S*. *pyogenes* [18], or Lbp, for laminin-binding protein [19], in *S*. *pyogenes* strains of different M types. Herein we chose to use the *lmb* denomination assigned to the M5005_Spy_1711 locus in strain MGAS5005 [17]. In summary, in streptococci the best-characterized components of the zinc uptake system are the *S*. *pneumoniae* AdcA and AdcAII lipoproteins whose orthologues in *S*. *pyogenes* are, respectively, AdcA, which shows 77% sequence conservation with the pneumococcal AdcA, and Lmb/Lsp/Lbp sharing 78% sequence homology with *S*. *pneumoniae* AdcAII. For both transporters the most conserved regions correspond to the domains identified as zinc-binding domains in the *S*. *pneumoniae* orthologues and, in particular, the metal-binding sites in Lbp and AdcAII are completely superimposable as determined by their structural characterization [15].

Null deletion mutants of the *adcA* and *lmb* genes were analysed for their capability to grow in zinc-depleted conditions. Expression of AdcA, Lmb and HtpA, the polyhistidine triad protein encoded by the gene adjacent to *lmb*, during growth under conditions of limited zinc availability was investigated by Western blot analysis in wild type and null mutant strains. Null mutants of *adcA* or *lmb* were more susceptible to zinc starvation, a phenotype that could be rescued by the addition of Zn^2+^ ions to the growth medium. In the wild type strain, AdcA was always present with little variation in expression levels between conditions of excess or limited zinc availability. In contrast, Lmb and HtpA were expressed at detectable levels only during growth in the presence of low zinc concentrations or in the null *adcA* mutant, where expression of *lmb* is required to compensate for the lack of *adcA* expression.

Hence, uptake of zinc in *S*. *pyogenes* strain MGAS5005 requires the action of both AdcA and Lmb only during growth in medium containing limiting amounts of Zn^2+^, whereas AdcA is always expressed and it possibly represents the main Zn^2+^ importer. Our results also suggest that these two SBPs must have partly redundant roles in zinc acquisition, as already described for AdcA and AdcAII in *S*. *pneumoniae* [8].

## Materials and Methods

### Ethics Statement

All animal studies were carried out in compliance with current Italian legislation on the care and use of animals in experimentation (Legislative Decree 116/92) and with the Novartis Animal Welfare Policy and Standards. Protocols were approved by the internal "Novartis Animal Ethical Committee" (approval number: AEC 200825) and authorized by the "Italian Ministry of Health" (authorization number: 21/2009-B).

### Bacterial strains and growth conditions

The *S*. *pyogenes* M1T1 serotype strain MGAS5005 is a clinical isolate carrying a frameshift mutation resulting in production of a truncated CovS, the sensor kinase element of a two-component signal transduction system which negatively regulates virulence [20]. Strain MGAS5005 and the isogenic mutants MGAS5005∆*adcA* and MGAS5005∆*lmb* were grown in Todd Hewitt broth (THB, Difco) supplemented with 5% of yeast extract (THY) or in tryptic soy agar (TSA, Difco) medium supplemented with 5% of defibrinated ram blood (TSA-blood). The complemented strain MGAS5005-∆*adcA*/pMU1328-*adcA* was grown in THY medium in the presence of 1 μg ml^-1^ of erythromycin.

The *Escherichia coli* strain DH10B (Invitrogen) carrying the pJRS233-∆*adcA* construct and strain HB101 (Promega) carrying the pJRS233-∆*lmb* construct were grown in Luria Bertani (LB) broth containing 200 μg ml^-1^ or 100 μg ml^-1^ of erythromycin, respectively, at 30°C or 37°C.

### DNA cloning

Deletion mutant strains were generated by allelic replacement using the temperature-sensitive shuttle vector pJRS233 (Perez-Casal *et al*., 1993). Suitable constructs were obtained by amplifying DNA fragments of 1 kb upstream and 1 kb downstream of the open reading frame of *adcA* or *lmb* using genomic DNA of strain SF370 as a template. The two 1 kb amplicons were joined in a single fragment by means of a SOEing PCR [21] and inserted between the *Bam*HI and *Xho*I restriction sites of vector pJRS233.

Complementation of the MGAS5005 *adcA* null mutant was obtained as follows. The chromosomal locus encompassing 143 nucleotides upstream of the *adcA* open reading frame, the *adcA* coding region and the downstream 51 nucleotides was amplified from genomic DNA of strain MGAS5005, generating a blunt PCR product of 1732 bp. This was ligated into the *Sma*I-digested pMU1328 expression vector [22]. The sequence of primers designed for all cloning experiments is given in Table 1.

**Table 1:** Bacterial strains, plasmids and primers used in this study.

**Strains**	**Relevant characteristics**	**Reference or source**
*E*. *coli*		
HB101	*supE*44 *hsdS*20 *(*r_B_^-^, m_B_^-^) *recA*13 *ara-*14 *proA*2 *lacY*1 *galK*2 *rpsL*20 *xyl-*5 *mtl-*1	Promega
DH10B	*hsdS* (r_B_^-^ m_B_^-^) *gal dcm*	Invitrogen
*S*. *pyogenes*		
MGAS5005	M1T1 serotype clinical isolate, sequenced strain	[13]
MGAS5005∆*adcA*	*adcA* null mutant of MGAS5005	This study
MGAS5005∆*lmb*	*lmb* null mutant of MGAS5005	This study
**Plasmids**	**Relevant characteristics**	**Reference**
pJRS233	temperature-sensitive shuttle vector used to create null mutants by allelic replacement, Amp^r^ Ery^r^	[20]
pMU1328	shuttle vector suitable for expression in streptococci, Ery^r^	[17]
**Primers**	**Sequence (5’-3’)**^a^	**Application**
P1forXhoI-adcA	GCGGCctcgagGAAGATTACCTTTGCTCAGCTGA	*adcA* null mutants
P2rev-adcA	GAAGATTTGCTTAGTGAGTTAAGAGATTCCTCCTTTGTTATTAACTG	
P3for-adcA	CAGTTAATAACAAAGGAGGAATCTCTTAACTCACTAAGCAAATCTTC	
P4forBamHI-adcA	GCGGCggatccTTCCTTGGTAGTGATAGCTGCAC	
P5for-adcA	GCCATTTAATACCATGGTGCC	
P6rev-adcA	CTGCAATCCTTAGGCGTTCTAA	
adcAko-ICfor	CCCAATCAATTTGGCATTGA	Amplification of ∆*adcA* locus
adcAko-ICrev	GTCATGGTTTCTTGCCATAA	
5’adcA-Bamfor	GCTAAggatccGCAACTGCTTAGCC	Cloning of *adcA* into pMU1328
3’adcA-Salrev	GCGGCgtcgacAAAGAAAAAGCAAACCTCCTTAAAAG	
lmb-ko-Salfor	CATCAACTGATTTCAgtcgacGG	*lmb* null mutants
lmb-ko-P2rev	CTTCAACTGTTGATAGAGCACACCTTTTTTCATAGTACCTCC	
lmb-ko-P3for	GGAGGTACTATGAAAAAAGGTGTGCTCTATCAACAGTTGAAG	
3'Spy1710-BglII	CGGTGTAGTagatctAAAAGTTC	
lmb-ko-ICfor	TGAATGAACCATTGTTTGCGAC	Amplification of ∆*lmb* locus
lmb-ko-ICrev	CCATGAGGCACCACATACCC	

a = restriction sites are indicated in lowercase and underlined.

The nucleotide sequences of PCR products, pJRS233 or pMU1328 constructs were determined using a BigDye Terminator V3.1 kit (Applied Biosystem) in an ABI PRISM 3700 Analyzer (Applied Biosystems).

### Deletion mutants of *adcA* and *lmb*

MGAS5005 competent cells were prepared as described by Sitkiewicz & Musser [23] or by Kimoto & Taketo [24]. MGAS5005-∆*adcA* and MGAS5005-∆*lmb* mutants were obtained as described by Perez-Casal *et al*. [25]. Briefly, after electroporation, GAS cells were plated onto TSA-blood + 0.5 μg ml^-1^ erythromycin medium and incubated at 30°C for episomal replication of the temperature-sensitive vector pJRS233. Colonies were screened for the presence of the SOEing-generated insert and the positive ones were inoculated into 3 ml of THY + 1 μg ml^-1^ erythromycin and grown at 37°C to force integration of the vector into the specific chromosomal locus. Excision from the chromosome of pJRS233 carrying the wild type allele of *adcA* or *lmb* was obtained by growing sequential dilutions of cultures in THY medium without erythromycin and incubating them overnight at 37°C. Allelic replacement was confirmed by PCR amplification of the specific locus using primers external to the chromosomal region encompassing the allelic exchange site. The PCR products were subsequently verified by sequencing.

### Growth in zinc depleted media

Bacterial cells were plated onto TSA-blood medium with the addition of 1 μg ml^-1^ erythromycin when required and incubated overnight at 37°C. A single colony was inoculated into 3 ml of THY and incubated overnight at 37°C. Each culture was then diluted 1:1000 into THY (with the addition of 1 μg ml^-1^ erythromycin when required) containing the appropriate concentration of the chelating agent Tetrakis-(2-Pyridylmethyl) ethylenediamine (TPEN, Sigma Aldrich) and also with the addition of ZnCl_2_ (Sigma-Aldrich). After incubation overnight at 37°C, growth was monitored spectrophotometrically at 600 nm. Experiments were performed in three independent replicates.

### Preparation of protein extracts

Bacterial cells were inoculated into 10 ml of the appropriate medium and incubated at 37°C until they reached an optical density reading of 0.4. The culture was harvested by centrifugation at 6,000 rpm for 5 min and washed with PBS. The bacterial pellet was then suspended in 500 μl of 10 mM Tris-HCl pH 8.0 containing 200 U ml^-1^ of mutanolysin (Sigma Aldrich) and 2 mg ml^-1^ of lysozyme (Sigma Aldrich) and incubated for 1 h at 37°C with shaking. After centrifugation for 5 min at 13,000 rpm, the pellet was suspended in 150 μl of 10 mM Tris-HCl pH 8.0, 1 mM EDTA pH 8.0, 2% SDS and mixed by vortexing for 30 sec. Samples were diluted 1:4 with the Dissolving Buffer (Invitrogen) in the presence of a reducing agent, boiled for 5 min and stored at -20°C until needed. Ten micrograms of each total cell extract were loaded onto a 4–12% Novex Bis-Tris NuPAGE pre-casted gel (Invitrogen) in MES buffer (Invitrogen) and stained with SimplyBlue SafeStain (Invitrogen).

### Western blot analysis

Five micrograms of total protein extracts were separated onto a 4–12% Novex Bis-Tris NuPAGE (Invitrogen) pre-casted gel and then transferred onto a nitrocellulose membrane with the iBlot apparatus (Invitrogen). Membranes were saturated with 10% skimmed milk in PBS-T buffer and probed with the appropriate antiserum, diluted in 1% skimmed milk in PBS-T buffer. Antisera against AdcA, Lmb and HtpA were raised in rabbits or mice immunised with purified recombinant proteins obtained as previously reported [26]. In our experimental conditions, the dilution used for each specific antiserum was 1:10,000 for rabbit anti-AdcA and 1:3,000 for mouse anti-Lmb or mouse anti-HtpA. After 1 h incubation at RT and 3 washes, an HRP-conjugated secondary antibody was added to the membrane at a 1:20,000 dilution in 1% skimmed milk PBS-T and incubated for 40 min under gentle agitation. The unbound antibody was washed away by 5 washes in the same buffer, the membrane was then overlaid with SuperSignal West Pico chemiluminescent substrate (Thermo Scientific Pierce) and exposed to a radiographic film. A marker for direct visualization of standard bands (MagicMark XP Western Protein Standard, Invitrogen) was used routinely for protein molecular mass estimation directly on Western blots. The antiserum specific for HtpA was found to give aspecific binding to a 37 kDa band present in all samples with an intensity that is directly correlated with the amount of total protein content of each sample, as evaluated by the Bradford method (Bio-Rad).

## Results and Discussion

### The Δ*adcA* mutant is more sensitive to zinc starvation

**Fig 1:**
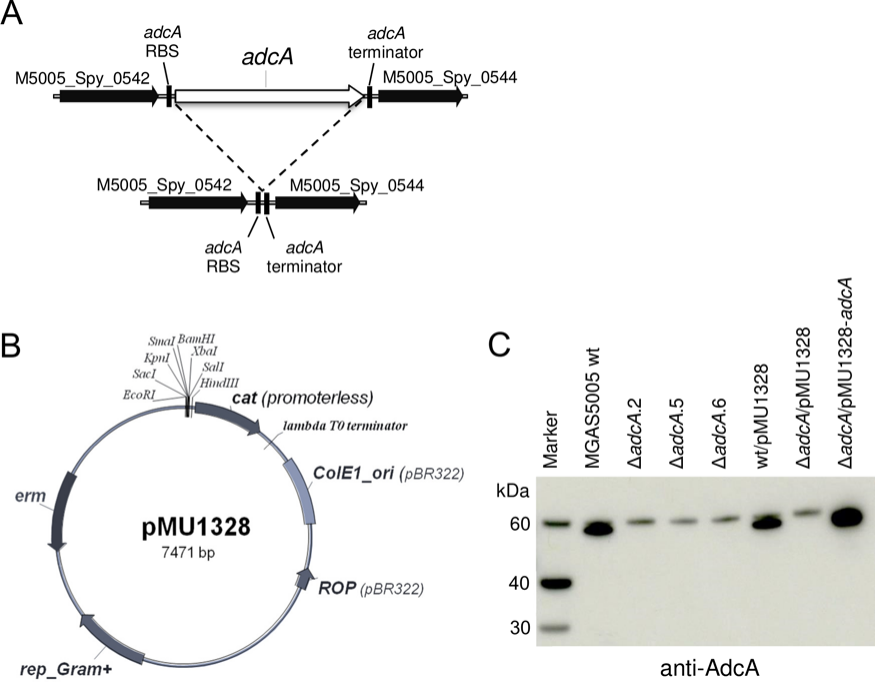
Construction of *adcA* null mutants. (**A**) Schematic representation of the *adcA* locus in S. *pyogenes* MGAS5005 wild type and Δ*adcA* deletion mutants. (**B**) Map of plasmid pMU1328 used for complementation of the Δ*adcA* deletion mutants. (**C**) Western blot analysis of AdcA expression in total cell extracts of wild type, three independent Δ*adcA* null mutants and a Δ*adcA* complemented with the pMU1328-*adcA* construct using anti-AdcA specific antibodies.

The gene product of *adcA* in the *S*. *pyogenes* strain MGAS5005 has been annotated as the orthologue of the high-affinity zinc uptake system protein ZnuA [17], known to be involved in zinc uptake both in Gram-negative [27] [28] and in Gram-positive bacteria [29]. Isogenic null mutants of *adcA* in strain MGAS5005 were generated as described in the Methods section by using the temperature-sensitive vector pJRS233 carrying the two regions flanking the *adcA* locus fused together to precisely delete the open reading frame assigned to AdcA (Fig 1A). One of the isogenic Δ*adcA* mutants was then used to obtain a strain complemented *in trans* by the wild type *adcA* gene carried on the expression vector pMU1328 [22]. The shuttle vector pMU1328, originally designed to identify promoter sequences in *Streptococcus sanguis*, *S*. *lactis* and *S*. *cremoris* [30], has been shown to be stably maintained in several Streptococcal species [31] [22]. Herein, we show that the pMU1328 replicon is suitable for the efficient expression of a homologous protein also in *S*. *pyogenes*. As is evident from Fig 1C, expression of AdcA was completely abolished in the Δ*adcA* mutant and approximately 2-fold higher than in the wild type strain when the same mutant was complemented with the pMU1328-*adcA* construct.

**Fig 2:**
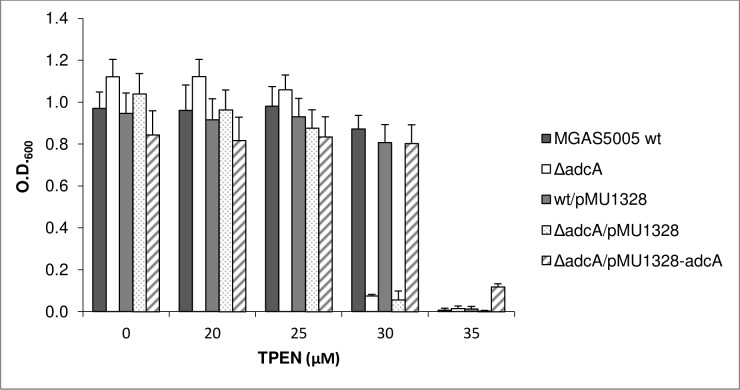
Susceptibility to Zn^2+^ starvation. Susceptibility to Zn^2+^ starvation of wild type, Δ*adcA* null mutant and complemented strains grown in THY medium containing increasing concentrations of the chelating agent N,N,N’N’-Tetrakis (2-pyridylmethyl)-1,2-ethylenediamine (TPEN). Error bars represent the standard deviation.

Since the gene product of *adcA* in other streptococci is known to be involved primarily in zinc uptake and homeostasis, we analysed the sensitivity to zinc starvation of the ∆*adcA* mutant and compared it with that of the wild type strain. The same mutant complemented with the pMU1328-*adcA* construct or carrying the empty pMU1328 vector were also included in our analysis as controls (Fig 2). The chelating agent N,N,N’,N’-Tetrakis-(2-pyridylmethyl)ethylenediamine (TPEN), a cell permeable high-affinity Zn^2+^ chelator used to reduce intra- and extra-cellular concentration of zinc in zinc homeostasis studies [32][16], was added at increasing concentrations to the growth medium of the ∆*adcA* mutant and MGAS5005 wild type strain. The Δ*adcA* mutant showed a higher susceptibility to zinc starvation. Growth was nearly abolished in the mutant by the addition of 30 µM TPEN, while it was only slightly decreased in the wild type or in the complemented strain (Fig 2). Complete inhibition of growth was observed at 35 μM TPEN for all strains, except for the Δ*adcA* mutant complemented with the pMU1328-*adcA* construct which showed some residual growth at 35 µM TPEN, possibly due to the higher level of AdcA expression in this strain (Fig 1C).

### Expression of Lmb and HtpA increases in Δ*adcA* mutants

**Fig 3:**
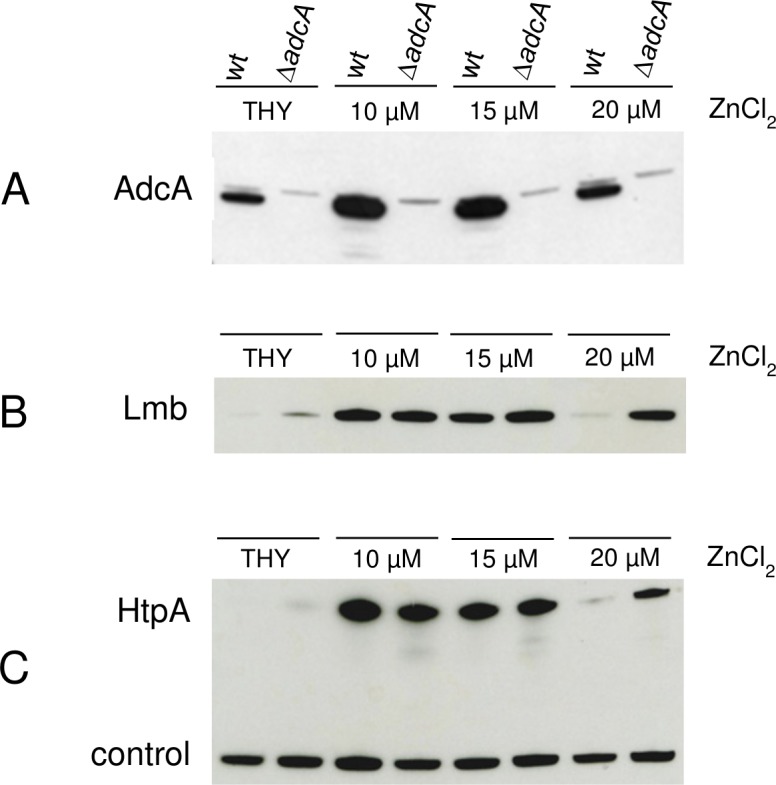
Expression of Lmb and HtpA in Δ*adcA* mutants. Western blot analysis of total cell extracts from *S*. *pyogenes* MGAS5005 wild type and Δ*adcA* null mutant grown in complete medium (THY) or in zinc-depleted medium (THY + 35 μM TPEN) containing increasing amounts of ZnCl_2_. (**A**) Western blot using anti-AdcA specific antibodies. (**B**) Western blot using anti-Lmb specific antibodies. (**C**) Western blot using anti-HtpA specific antibodies.

The increased susceptibility to zinc starvation of the *adcA* null mutant strongly supports the notion that AdcA is indeed involved in zinc homeostasis in *S*. *pyogenes*, as already reported for other streptococci. In particular, in *S*. *pneumoniae* a recent characterization of AdcA has highlighted its complementarity in zinc homeostasis with AdcAII, the other Zn^2+^-binding SBP of streptococci, and demonstrated the cooperative functionality between the two importers [8]. Furthermore, immediately downstream of the gene coding for the AdcAII homologue Lmb in all the GAS strains characterized to date is located the gene coding for the histidine triad protein HtpA, a member of a family of proteins containing multiple copies of a histidine triad motif known to bind Zn^2+^ ions [33][34]. The gene coding for HtpA is highly conserved both in sequence and location also in GBS strains [33] and in *S*. *pneumoniae*, where *adcAII* is also located in the same operon as *phtD*, one of the four genes coding for a pneumococcal histidine triad protein [34][35]. For these reasons, we analysed the differences in expression levels of Lmb and HtpA in the Δ*adcA* mutant grown in zinc-depleted medium supplemented with increasing amounts of zinc ions (Fig 3). Firstly, it should be noted that in the wild type strain expression of AdcA was highest in medium supplemented with 10–15 μM of zinc ions, while at 20 μM ZnCl_2_ it was comparable with the level of expression observed in complete medium (Fig 3A). This suggests that limiting amounts of Zn^2+^ ions are able to induce only a small increase in expression of AdcA. On the contrary, Lmb and HtpA were expressed at significantly higher levels in both wild type and Δ*adcA* strains grown in the presence of 10–15 μM Zn^2+^ ions (Fig 3B and 3C). During growth in rich medium containing excess zinc ions (THB), expression of Lmb and HtpA was undetectable in wild type cells and low in the Δ*adcA* mutant. Moreover, Lmb and HtpA were expressed at higher levels in the Δ*adcA* mutant grown in the presence of 20 μM Zn^2+^ ions, a growth condition that presumably requires a functional AdcA for zinc homeostasis, which in a Δ*adcA* mutant could only be provided by an increased expression of Lmb and, consequently, of HtpA.

### The Δ*lmb* mutant is as sensitive to zinc starvation as the Δ*adcA* strain

**Fig 4:**
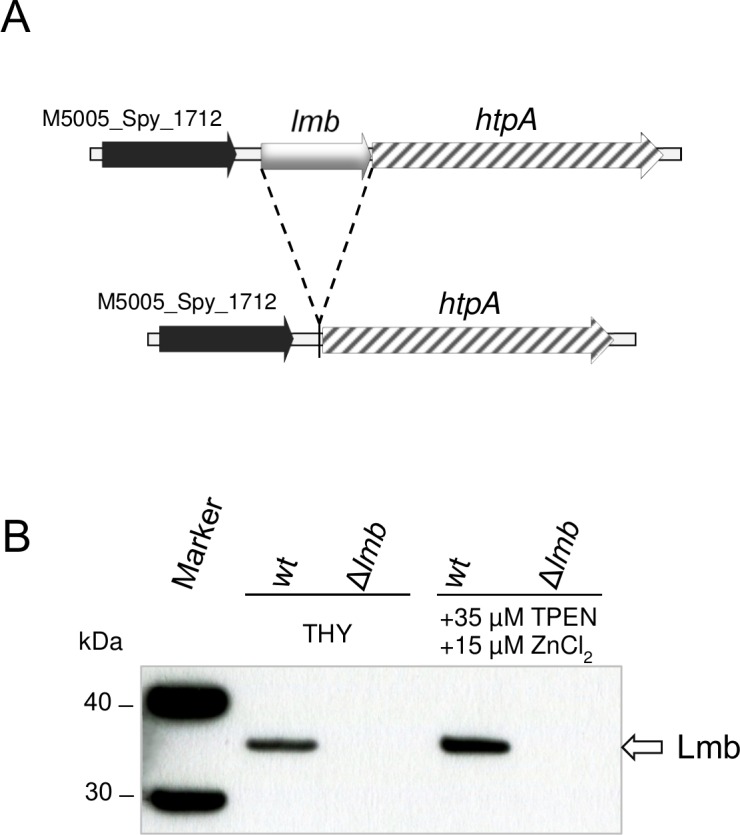
Construction of Δ*lmb* null mutants. (**A**) Schematic representation of the *lmb* locus in S. *pyogenes* MGAS5005 wild type and Δ*lmb* deletion mutants. (**B**) Western blot analysis of Lmb expression using anti-Lmb specific antibodies in total cell extracts of wild type and of a Δ*lmb* null mutant grown in THY medium or in THY with the addition of 35 μM TPEN and 15 μM ZnCl_2_. The latter growth medium was tested since it was observed to give higher expression of Lmb in our experimental conditions.

An in-frame deletion mutant of *lmb*/*lsp* has already been described in the *S*. *pyogenes* strain HSC5 [36] and shown to have a role in zinc homeostasis and pathogenesis [16]. However, due to the variability in genetic composition between different *S*. *pyogenes* strains [37] and the complexity of zinc homeostasis control in any biological system, we generated a Δ*lmb* mutant in strain MGAS5005 in order to investigate the contribution of Lmb to zinc uptake in the same genetic background used for characterizing AdcA. The map of the locus in the *lmb* null mutant is outlined in Fig 4A and the Western blot analysis carried out to confirm the absence of Lmb expression in the mutant strain is given in Fig 4B.

**Fig 5:**
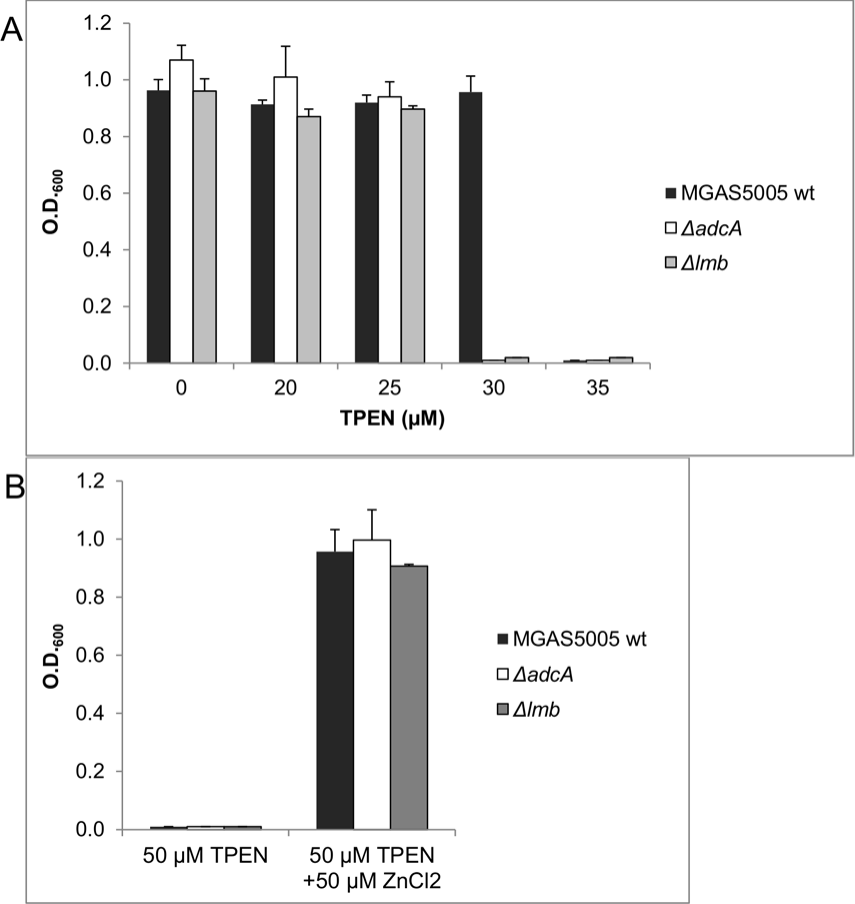
Inhibition of growth by TPEN is rescued by the addition of Zn^2+^ ions. (**A**) Susceptibility to Zn^2+^ starvation of wild type, Δ*adcA* and Δ*lmb* null mutants grown in THY medium containing increasing concentrations of the chelating agent TPEN. (**B**) Inhibition of growth by 50 μM TPEN is rescued by the addition of either 50 μM Zn^2+^ in wild type as well as in Δ*adcA* and Δ*lmb* null mutants. Error bars represent the standard deviation.

Loss of *adcA* or *lmb* expression in the two null mutants did not significantly impair their growth rates in THY medium with respect to that of the wild type strain (S1 Fig). Furthermore, growth in the presence of increasing concentrations of TPEN, for both Δ*adcA* and Δ*lmb* mutants, was also comparable with that of the wild type strain at concentrations up to 25 μM TPEN. Conversely, at 30 μM TPEN only the wild type strain was able to grow and at 35μM TPEN growth was completely inhibited for all strains (Fig 5A). Addition of equimolar amounts of Zn^2+^ ions to the zinc-depleted medium could rescue growth of both Δ*adcA* and Δ*lmb* mutant strains (Fig 5B), thus confirming that in *S*. *pyogenes* these two SBPs have overlapping roles in zinc acquisition, as already described in *S*. *pneumoniae* [8].

**Fig 6:**
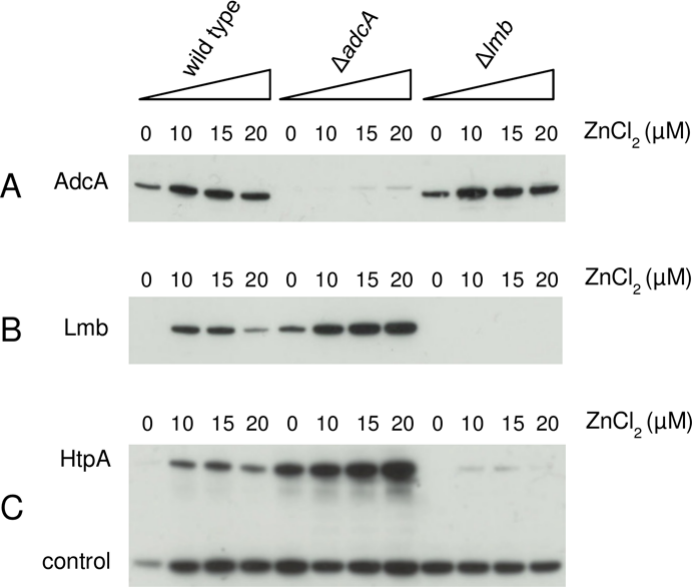
Expression of AdcA, Lmb and HtpA during growth in zinc-depleted medium. Western blot analysis of total cell extracts from *S*. *pyogenes* MGAS5005 wild type, Δ*adcA* and Δ*lmb* null mutants grown in complete medium (THY) or in zinc-depleted medium (THY + 35 μM TPEN) containing increasing amounts of ZnCl_2_. (**A**) Western blot using anti-AdcA specific antibodies. (**B**) Western blot using anti-Lmb specific antibodies. (**C**) Western blot using anti-HtpA specific antibodies.

Wild type *S*. *pyogenes* MGAS5005 and isogenic mutant strains lacking *adcA* or *lmb* were analysed for expression of AdcA, Lmb and HtpA during growth in complete medium (THY) or in zinc-depleted medium (THY + 35 μM TPEN) containing increasing amounts of ZnCl_2_. The immunoblot analyses presented in Fig 6 confirm the results obtained for the Δ*adcA* mutant (Fig 3). Lmb and HtpA were both expressed at higher levels in the Δ*adcA* mutant, strongly supporting the assumption that also in *S*. *pyogenes* AdcA and Lmb have overlapping roles in zinc acquisition [8] (Fig 6B and 6C).

An additional outcome of this analysis is the observation that expression of Lmb and HtpA was not detectable in the wild type strain grown in THY (lanes 0 in Fig 6B and 6C). As this condition probably represents growth in the presence of excess zinc ions, we can conclude that the main importer of zinc ions in *S*. *pyogenes* is AdcA. In fact, AdcA is always expressed in wild type cells, with little variation in the level of expression during growth in media with a different content of Zn^2+^ ions or in the absence of a functional Lmb, as in the case of the Δ*lmb* mutant (Fig 6A). In contrast, Lmb may represent the zinc importer expressed only during growth in zinc-depleted media while, in the absence of AdcA as in the Δ*adcA* mutant, it needs to be overexpressed only during growth in an excess of Zn^2+^ ions. This implies that in normal conditions, when the concentration of Zn^2+^ ions is 20 μM or higher, AdcA is sufficient to maintain a physiological homeostasis of zinc ions, while in zinc-depleted growth conditions both importers are required for the maintenance of a correct influx of zinc ions.

Conversely, an excess of intracellular free zinc ions is known to be highly toxic, thus the need to overexpress both Lmb and HtpA in the Δ*adcA* mutant may be due to the different zinc-binding capabilities of AdcA and Lmb. Indeed, a recent characterization of AdcA and AdcAII (Lmb) of *S*. *pneumoniae* has shown that, as previously reported for the orthologous SBPs in other bacteria [7] [38] [39], AdcA has two zinc-binding domains [8] while AdcAII contains only one zinc-binding site [9]. Although the affinity and stoichiometry of zinc binding to AdcA and Lmb in *S*. *pyogenes* have not been determined, it is reasonable to assume that they are similar to those reported for other streptococci during growth *in vitro*. Thus, if AdcA and Lmb contribute to the maintenance of a low intracellular content of free Zn^2+^ ions through their capability of binding zinc, expression of Lmb should increase at least two-fold in the absence of AdcA in order to provide the correct number of zinc binding sites. In fact, a greater than three-fold increase of Lmb expression was observed in the Δ*adcA* mutant, particularly during growth in the presence of zinc ions in excess (Fig 6B). Interestingly, the increase of HtpA expression was even higher in the same conditions (Fig 6C). These variations in level of expression are confirmed by the quantitative analysis presented in S2 Fig.

As the function of *S*. *pyogenes* HtpA has yet to be clarified, we propose that, by analogy with the role described for the pneumococcal histidine triad proteins [40][35][10], the main task of HtpA is to facilitate Zn^2+^ acquisition exclusively through Lmb. Indeed, since the null *lmb* mutant obtained in this work also lacked expression of HtpA (Fig 6C), but could grow at a normal rate and with levels of AdcA expression very similar to those observed in the wild type strain grown in the same conditions (Fig 6A), it may be inferred that acquisition of Zn^2+^ ions through AdcA does not require HtpA. Whether AdcA is capable of binding zinc unaided or needs the cooperation of another histidine triad protein remains to be investigated. Future work will be focused on studying the interplay between the different SBPs involved in zinc ion uptake and the histidine triad proteins that presumably contribute to the maintenance of zinc homeostasis in *S*. *pyogenes*.

## Supporting Information

S1 Fig Growth curve of *S*. *pyogenes* wild type strain MGAS5005 and its isogenic Δ*adc* and Δ*lmb* mutants. Strains were grown in THY medium and the increase in cell density over time was measured spectrophotometrically. Overnight cultures were diluted at a ratio of 1:2 in fresh THY and grown in a static environment for 2 hours. The growing cells were then diluted to an OD_600_ of 0.03 in fresh THY. The cells were statically grown in a microaerobic environment and the OD_600_ of the culture was measured at regular intervals.
(TIF)

S2 Fig Expression of AdcA, Lmb and HtpA during growth in zinc-depleted medium. Relative intensities of the bands detected in Western blot analysis of total cell extracts from *S*. *pyogenes* MGAS5005 wild type, Δ*adcA* and Δ*lmb* null mutants grown in complete medium (THY) or in zinc-depleted medium (THY + 35 μM TPEN) containing increasing amounts of zinc ions. The relative intensities were determined on the basis of two independent experiments using the Phoretix 1D software (Cleaver Scientific Ltd., Rugby, United Kingdom). The data were normalised on the control bands of each experiment (see Fig 6). Error bars represent the standard deviation.
(TIF)
