# Supplementary figures and images for: Zn2+ Uptake in Streptococcus pyogenes: Characterization of adcA and lmb Null Mutants
Source: PLoS One. 2016 Mar 31;11(3):e0152835. doi: 10.1371/journal.pone.0152835 (PMC4816340; doi:10.1371/journal.pone.0152835)

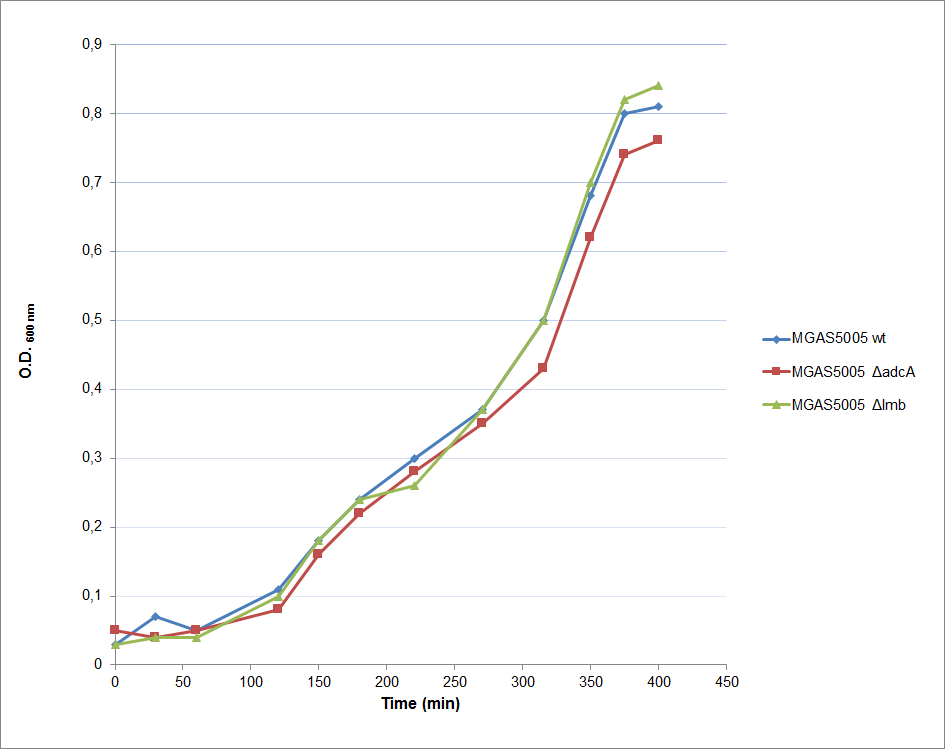

Supplement: S1 Fig — Strains were grown in THY medium and the increase in cell density over time was measured spectrophotometrically. Overnight cultures were diluted at a ratio of 1:2 in fresh THY and grown in a static environment for 2 hours. The growing cells were then diluted to an OD600 of 0.03 in fresh THY. The cells were statically grown in a microaerobic environment and the OD600 of the culture was measured at regular intervals. (TIF) [file pone.0152835.s001.tif]

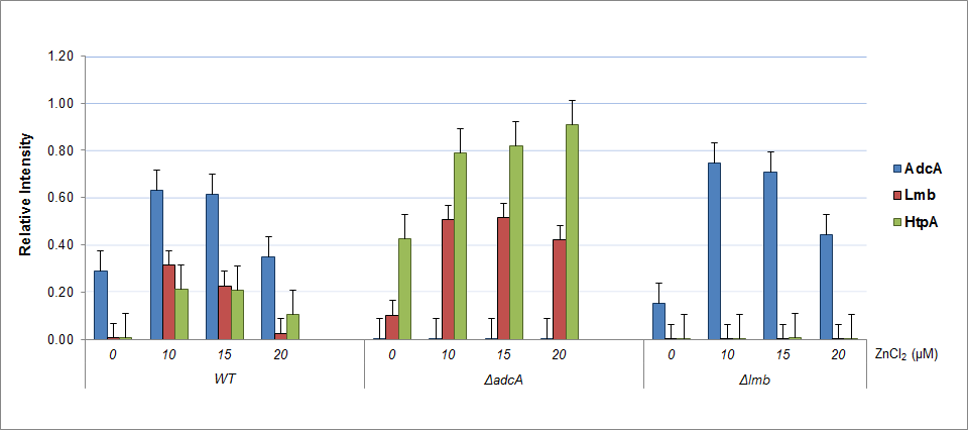

Supplement: S2 Fig — Relative intensities of the bands detected in Western blot analysis of total cell extracts from S. pyogenes MGAS5005 wild type, ΔadcA and Δlmb null mutants grown in complete medium (THY) or in zinc-depleted medium (THY + 35 μM TPEN) containing increasing amounts of zinc ions. The relative intensities were determined on the basis of two independent experiments using the Phoretix 1D software (Cleaver Scientific Ltd., Rugby, United Kingdom). The data were normalised on the control bands of each experiment (see Fig 6). Error bars represent the standard deviation. (TIF) [file pone.0152835.s002.tif]
